# Comparison of the Impact of NaIO_4_-Accelerated, Cu^2+^/H_2_O_2_-Accelerated, and Novel Ion-Accelerated Methods of Poly(l-DOPA) Coating on Collagen-Sealed Vascular Prostheses: Strengths and Weaknesses

**DOI:** 10.1021/acsami.4c05979

**Published:** 2024-07-24

**Authors:** Michał Fornal, Agnieszka Krawczyńska, Anna Belcarz

**Affiliations:** † Chair and Department of Biochemistry and Biotechnology, Medical University of Lublin, Chodźki 1, 20-093 Lublin, Poland; ‡ Faculty of Materials Science and Engineering, Warsaw University of Technology, 141 Wołoska, 02-507 Warsaw, Poland

**Keywords:** catecholamine polymerization acceleration, mono-/divalent ions, hydrogen peroxide, periodate, biological activity, vascular grafts

## Abstract

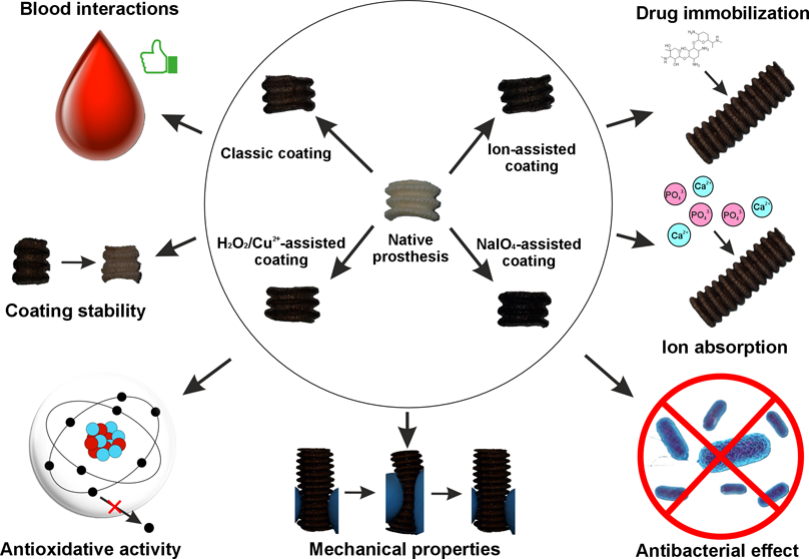

Sensitive biomaterials subjected to surface modification require delicate methods to preserve their structures and key properties. These include collagen-sealed polyester vascular prostheses. For their functionalization, coating with polycatecholamines (PCAs) can be used. PCAs change some important biological properties of biomaterials, e.g., hydrophilicity, bioactivity, antibacterial activity, and drug binding. The coating process can be stimulated by oxidants, organic solvents, or process conditions. However, these factors may change the properties of the PCA layer and the matrix itself. In this work, collagen-sealed vascular grafts were functionalized with a poly(l-DOPA) (PLD) layer using novel seawater-inspired ion combination as an accelerator, compared to the sodium periodate, Cu^2+^/H_2_O_2_ mixture, and accelerator-free reference methods. Then, poly(l-DOPA) was used as the interface for antibiotic binding. The coated prostheses were characterized (SEM, FIB-SEM, FTIR, UV/vis), and their important functional parameters (mechanical, antioxidant, hemolytic, and prothrombotic properties, bioactivity, stability in human blood and simulated body fluid (SBF), antibiotic binding, release, and antibacterial activity) were compared. It was found that although sodium periodate increased the strength and drug-binding capacity of the prosthesis, it also increased the blood hemolysis risk. Cu^2+^/H_2_O_2_ destabilized the mechanical properties of the coating and the graft. The seawater-inspired ion-accelerated method was efficient, stable, and matrix- and human blood-friendly, and it stimulated hydroxyapatite formation on the prosthesis surface. The results lead to the conclusion that selection of the PCA formation accelerator should be based on a careful analysis of the biological properties of medical devices. They also suggest that the ion-accelerated method of PLD coating on medical devices may be highly effective and safer than the oxidant-accelerated coating method.

## 1 Introduction

Polydopamine (PDA), belonging to polycatecholamines (PCAs), is a major pigment present in natural melanin (eumelanin).^1^ Due to its extreme adhesiveness to different surfaces in aqueous media, PDA has been used for functionalization of a wide range of materials since 2007.^2^ Formation of PDA coating, as a low-cost, single-step, and environment-friendly method, is nowadays a frequently used method for modification of graphene nanosheets,^3,4^ Fe_3_O_4_ nanoparticles (for drug delivery, for catalyst support, adsorbents, and sensors),^4−6^ silica nanoparticles,^7^ polymers,^8,9^ metals,^10^ and many other matrices. Moreover, the coatings made of PDA and dopamine-related derivatives show beneficial properties, which may positively alter the properties of coated materials. For example, they provide photoprotective ability, exhibit antioxidant and metal-chelation properties, absorb ultrasonic sound waves, change the hydrophilicity of surfaces, increase the adhesiveness of many eukaryotic cells and bioactivity in simulated body fluid (SBF) (the property important for bone replacement materials), act as antibacterial surfaces, produce heat under IR stimulation, and show many other functions.^11−13^ With the intensification of research, it was found that other catecholamines, including norepinephrine and levodopa (l-DOPA), form efficient coatings on various matrices.^14^ Moreover, polycatecholamine coatings allow for secondary coupling reactions with different organic molecules containing free amino or thiol groups. This mechanism is possible due to the presence of catechol domains, which can react with thiols and amines via Michael addition or Schiff base reactions.^15,16^ This opens the possibility to modify the coated materials with antibacterial and anti-inflammatory drugs, growth factors, hemostatic agents, etc. Therefore, enormous interest in this technique is observed.

PCAs require mild reaction conditions for their formation.^17^ Slightly alkaline (approximately 8.0) pH, room temperature, and presence of dissolved oxygen are sufficient to allow this process.^2^ In light of this knowledge, PCAs seem to be the preferred modification for sensitive matrices, such as knitted polyester vascular grafts sealed with collagen. Such grafts are commonly used in vascular surgery for blood vessel replacement. Unfortunately, when implanted, they may undergo bacterial adhesion due to protein content, which is often followed by biofilm formation. This creates the problem of prosthesis infection, which still remains critical in modern vascular surgery because despite its low rate (it occurs in 0.6–5% of patients after reconstructions at the aorto-iliac level), the mortality in this group is high (25–88%). Worse, 40–70% of the patients need to undergo major amputation. The cost related to vascular graft infection is estimated to be $640 million annually only in the United States.^18^ Reduction of both social and financial costs of this problem could therefore result in an increased interest in methods of modifying prostheses to protect them against bacterial colonization.

The strategies used to functionalize protein-sealed vascular grafts are few due to, among others, the sensitivity of proteins to modification factors. The simplest method is physical adsorption of active molecules to the prostheses by impregnation. This technique does not affect the structure of the sealant (collagen, gelatin, albumin), but drug-impregnated prostheses usually release the active molecules relatively quickly (within hours). This mechanism was used to create prostheses impregnated with rifampicin, triclosan, and silver acetate.^19^ Another method, based on drug-to-protein covalent binding, requires the glutaraldehyde-based activation of the protein with subsequent coupling of an antibiotic containing a free amino group (Schiff base formation). This method was found efficient for gentamicin bonding to gelatin-sealed poly(ethylene terephthalate) vascular prostheses.^20^ However, glutaraldehyde exhibits relatively strong toxicity, and its remains must be carefully eluted from the grafts to avoid side effects. There are several other proposals for functionalization of cardiovascular materials. For example, polycarboxybetaine coatings were proposed for spin-coating modifications of cardiovascular implants, which can be coupled with active peptides (e.g., Arg-Glu-Asp-Val peptide) due to the content of carboxy groups via NHS/EDC chemistry.^21^ However, it requires a multistep modification procedure and organic solvents for deposition of the coating. Knitted polyester vascular prostheses can also be modified with methyl-β-cyclodextrin for further adsorption of ciprofloxacin;^22^ however, the fixation of dextrin derivative on the graft requires elevated temperatures (150–180 °C), which would be devastating for the collagen sealing layer. Summarizing, all of the methods listed above exhibit short-term antibacterial efficacy, produce potentially toxic matrices, require many steps, or are time-consuming. Moreover, none of the mentioned methods allows for the functionalization of the modified matrices in a more complex way, taking into consideration their photoprotective, antioxidant, antibacterial, and metal-chelation properties, altered surface hydrophilicity, IR-to-heat transformation, and other specific functions, in contrast to PCA coatings (as mentioned above).

Considering the state-of-the-art, vascular graft coating with PCA, potentially further coupled with antibiotics containing a free amino group, seems to be a promising alternative for protecting grafts against bacterial infection while preserving the structure and functionality of polyester fibers and the collagen sealing layer. It should be noted that PDA deposition on vascular implants for their functionalization has already been investigated. According to available literature, popular matrices such as polyurethane, stainless steel, silicon, titanium wafers, expanded poly(tetrafluorethylene) and poly(l-lactide-*co*-ε-caprolactone) as well as zinc were used to verify these assumptions.^23−27^ In most of these studies, PDA coating was used to immobilize the vascular endothelial growth factor (VEGF^24,26^), gelatin,^27^ or copper.^23^ The expected effects of the modification were increased nitric oxide synthesis, increased biocompatibility and hemocompatibility, reduction of neointima formation and macrophage infiltration, rapid endothelialization, and increased neovascularization in surrounding tissues.^23−27^ However, to our knowledge, no attempts have been made to immobilize PCA on one of the currently most popular vascular grafts, namely, knitted polyester grafts sealed with collagen. Besides, the enhancement of the PCA coating on vascular grafts has not been taken into account so far.

Enhancement of PCA formation increases not only the rate of polymerization but also the thickness of the coating layer.^28,29^ Thicker PCA coating may also increase the quantity of catechol-bound active molecules, e.g., antibiotics, thus increasing the potential antibacterial activity of such modified vascular grafts and reducing the infection-related devastating consequences for human recipient’s health. Polymerization of catecholamines can be accelerated under the reaction conditions. Organic solvents, as alcohols or piperidine, are helpful to induce PDA coating of hydrophobic surfaces.^30,31^ Nonchemical treatment may also affect PDA formation. For example, hydrothermal process by autoclaving, microwave treatment, or use of plasma-activated water may trigger PDA polymerization even in the absence of oxidants.^32−34^ Organic molecules of oxidative properties (e.g., laccase) can also mediate the PDA coating.^35^ Chemical oxidants induce PDA polymerization also in acidic pH (when oxygen is unavailable as an oxidant of the catechol group to form its intermediate, catechol quinone). Sodium periodate in alkaline pH increased the polymerization rate of dopamine.^28^ CuSO_4_ in combination with H_2_O_2_ was found to induce rapid deposition of PDA coatings on solid surfaces.^29^ However, PDA-accelerating factors can strongly affect the final properties of the matrix. For example, laccase used for PCA polymerization not only accelerated this process but also was incorporated within PDA coating, providing the unique biocathode for glucose/O_2_ biofuel units.^35^ The high concentration of sodium periodate used for PDA formation increased the matrix hydrophilicity because of degradation of quinone units with the simultaneous formation of carboxyl groups (sodium periodate, similar to H_2_O_2_, is a relatively strong oxidant).^28^ Therefore, for vascular prostheses coated with PCAs for further antibiotic immobilization, an appropriate method should be selected to enable the binding of the maximum amount of the drug and simultaneously to preserve the critical physicochemical and therapeutic properties of the prosthesis itself. This precaution is crucial for the design of all modified biomaterials because structural and physical properties of tissue constructs are expected to differentially affect the cell–biomaterial interactions. Among others, pore network, minimal feature size, mechanical properties, swelling, and surface features can influence the biological outcomes, including cytotoxicity, viability, proliferation and differentiation, adhesion, alignment, and signaling of the cells.^36^ The relation between structural characteristics of collagen-polyester vascular prostheses and their good performance in clinical applications was reported for Omniflow graft (Bio Nova International, Australia^37^). Authors of a recent review concerning constructional design components affecting the mechanical response and cellular activity of vascular grafts strongly emphasized on determining their mechanical characteristics, including suturability, compliance, tensile strength, burst pressure, and blood permeability.^38^ Therefore, preservation of structural integrity is extremely important for the durable functionality of vascular grafts.

A new PCA formation-accelerated method inspired by the composition of seawater may fulfill these requirements. Recently, we published the pilot results of collagen-sealed vascular prosthesis coating with PCAs using a buffer supplemented with two main ions of seawater: Mg^2+^ and Na^+^. The ions were used in concentrations as in seawater, with and without the copresence of Cu^2+^.^39,40^^39,40^ This simple ion combination caused a 3-fold increase of the gentamicin-binding capacity of the coated prostheses, in comparison with the ion-free buffer.^39^ Moreover, the drug binding was intensified when l-DOPA was used instead of dopamine, forming poly(l-DOPA) (PLD) coating.^39,40^ Simultaneously, the very mild conditions of this modification seemed to be less harmful to such sensitive matrices when compared with the chemical oxidant-accelerated process.

To verify this hypothesis, the optimization of the ion-accelerated method was performed for collagen-sealed vascular prosthesis modification, with gentamicin-binding capacity as a criterion. Gentamicin was selected for this purpose because its molecule contains free amino groups. Then, the selected physicochemical and biocompatibility parameters of the prostheses coated by PLD using the ion-accelerated method were evaluated to check their possible impact on the graft properties. In the experiments, the prostheses modified by the ion-accelerated method were compared with those modified by the sodium periodate-accelerated method and the Cu^2+^/H_2_O_2_-accelerated method (as methods previously reported in the literature as highly effective in formation of a thick PDA layer).

## 2 Materials and Methods

### 2.1 Modification of Prosthesis

FlowNit Bioseal collagen-coated knitted polyester grafts (ø60 mm, 600 mm) were purchased from JOTEC GmbH, Hechingen, Germany. Vascular prostheses were coated with PLD by a one-step deep coating method. Briefly, prosthesis fragments (50 ± 2 or 100 ± 2 mg) were immersed in 10 mM Tris buffer pH 8.5 containing 2 mg/mL l-DOPA (Sigma) and incubated for 24 h using a roller mixer RM5-30V (CAT, Germany). The stimulants of PLD polymerization were a complex of several ions (for P–C–I samples), sodium periodate (for P–C–P samples), Cu^2+^/H_2_O_2_ (for P–C–H samples), and no stimulants in reference (for P–C samples). For P–C–I samples, the concentrations of selected ions were first optimized. On the basis of reports concerning the composition of seawater from different sea areas,^41−46^ the average values of the selected ion concentration were set as follows: 472.2 mM for Na^+^, 53.7 mM for Mg^2+^, 10.6 mM for Ca^2+^, and 28.2 mM for SO_4_^2–^. Moreover, on the basis of earlier reports,^28^ a 0.5 mM Cu^2+^ concentration was additionally used. To verify to what extent the seawater-specific ion concentration is crucial for PCA polymerization, each ion concentration was increased or decreased 10-fold and also tested. 20 mM NaIO_4_ for P–C–P sample preparation and 19.6 mM H_2_O_2_ + 0.5 mM Cu^2+^ for the synthesis of P–C–H samples were chosen according to the procedures cited elsewhere.^28,29^ Temperature, shaking speed, and pH were optimized for P–C–I, P–C–P, P–C–H, and P–C samples within the ranges 20–50 °C, pH 5.5–8.5, and 10–30 rpm. After PLD coating, graft pieces were rinsed several times with DI water to remove nonbound PLD and l-DOPA and dried at 37 °C. For comparison, control grafts (P) were rinsed in pure 10 mM Tris buffer at pH 8.5 and washed several times in DI water, followed by drying at 37 °C.

### 2.2 Characterization of Prosthesis

UV–vis absorption spectra of the PLD polymerization process were collected in quartz cuvettes (Starna Scientific Ltd., U.K.) within the range 190–700 nm, with pure 10 mM Tris buffer pH 8.5 as a reference, using a Genesys 10s UV–vis spectrophotometer and VisionLite software (Thermo Scientific). The colors of the reaction mixture were visualized using an Olympus E-520 camera (Olympus, Germany). FTIR-ATR spectra were collected using a Vertex 70 spectrometer equipped with ATR-diamond crystal accessory (Bruker), with resolution 4 cm^–1^ and 64 scans per spectrum. Samples were dried before analysis at 37 °C for 24 h to remove the unbound water. The spectra were then analyzed using OPUS 7.0 software (Bruker, Billerica, MA) to calculate the ratios between the 1233 cm^–1^ band and the 1454 cm^–1^ band. Characterization of prosthesis samples (after the synthesis, incubation in blood, incubation in SBF, and after the bacterial adhesion test) was performed using a scanning electron microscope (SEM) Su8000 Hitachi at the accelerating voltage of 3 kV. A focused ion beam (FIB) NB5000 Hitachi was applied to prepare and visualize the cross-sectional structure of PLD-coated collagen-sealed prostheses. Before SEM and FIB observations, samples were covered with Au of 10 nm thickness. SEM and FIB observations were performed at the Faculty of Materials Science and Engineering at the Warsaw University of Technology. Mechanical parameters of the modified prostheses were performed on 30 mm-long fragments of prostheses of an original tube-like shape, previously soaked in 0.1 M phosphate buffer pH 7.4, to mimic the operating conditions, in triplicate. The compression test was carried out using the EZ Test EX-SX universal testing machine (Shimadzu, Kyoto, Japan) equipped with the Trapezium program and a force sensor of 100 N, with a crosshead speed of 5 mm/min, starting after obtaining a force value of 0.05 N to eliminate gaps between the sample and the grips. The mechanical compression was carried out until 50% compression was reached. Then, the crosshead returned to the starting point, and the cycle was repeated 5 times. The stress detected at 50% graft compression was evaluated.

### 2.3 Stability of PLD Coatings in Buffers of Different pHs, in SBF and Human Blood

For stability in buffers and SBF, 50 ± 2 mg of modified prostheses was incubated in the wells of 24-well plates (Nest Biotechnology, China), either in 1 mL of 0.1 M Britton–Robinson buffer pH 2–12 for 7 days or in 1 mL of SBF pH 7.42, for 28 days, at 37 °C and 100 rpm, in an Innova 42 incubator (New Brunswick Scientific, Edison, NJ). After that, absorbance values of both buffers and SBF were measured at 280 nm, using a Synergy H4 Hybrid microplate reader (BioTek). Images of both buffers and SBF in well plates as well as prostheses samples after the incubation were taken using an Olympus E-520 camera (Olympus, Germany). Color change of the prostheses samples was calculated using ImageJ 1.52v software. For stability in blood, 50 ± 2 mg of prostheses samples was incubated in a 24-well plate containing 1 mL of citrated human blood (collected from a healthy volunteer, after approval from the Bioethical Committee of Medical University of Lublin, No. KE-0254/114/04/2023), supplemented with a 1% antibiotic antimycotic solution (Merck), to prevent microorganism growth, at 37 °C and 100 rpm for 72 h, in an Innova 42 incubator (New Brunswick Scientific, Edison, NJ). Then, the samples were washed with phosphate buffer, pH 7.4, to remove elements of blood. The color of the samples before and after incubation was compared on the basis of images collected with an Olympus E-520 camera (Olympus, Germany). Color intensity of the samples was calculated using ImageJ 1.52v software.

### 2.4 Antioxidative Properties

#### 2.4.1 DPPH Test

Prostheses samples (50 ± 2 mg) were immersed in 1 mL of DI water and an equal volume of 0.2 mg/mL DPPH (2,2-diphenyl-1-picrylhydrazyl, Sigma) in an ethanol (POCH, Poland) solution. Absorbance of the solution after 10 min of incubation at 30 °C in DTS-4 (ELMI, Newbury Park, CA) was taken using a Synergy H4 Hybrid microplate reader (BioTek), at λ = 515 nm. The control sample consisted of the same mixture without prostheses. After the measurement, the prosthesis pieces were washed in DI water and dried, and the measurement was repeated. Statistically significant differences between the control and prostheses samples were considered at *p* < 0.05, according to a one-way ANOVA with post hoc Dunnett’s test (GraphPad Prism 8.0.0 Software, San Diego, CA). ABTS test: The reagent was obtained by dissolving 7.4 mM ABTS (2,2′-azino-bis(3-ethylbenzothiazoline-6-sulfonic acid); Sigma) and 2.6 mM potassium persulfate (Sigma) in MQ water. After 16 h, the mixture was diluted to Abs_734 nm_ = 0.7 using phosphate buffer pH 7.4. Prosthesis samples (50 ± 2 mg) were immersed in 2 mL of ABTS reagent. Samples were incubated at 30 °C in a DTS-4 shaker (ELMI, Newbury Park, CA) for 10 min. Absorbance of the solution was taken using a Synergy H4 Hybrid microplate reader (BioTek), at λ = 734 nm. The control sample consisted of the same mixture without prostheses. After the measurement, the prosthesis pieces were washed in DI water and dried, and the measurement was repeated. Statistically significant differences between the control and prostheses samples were considered at *p* < 0.05, according to a one-way ANOVA with post hoc Dunnett’s test (GraphPad Prism 8.0.0 Software, San Diego, CA).

### 2.5 Prosthesis Interactions with Human Blood

For the tests, citrated human blood (collected from healthy volunteers, after approval from the Bioethical Committee of Medical University of Lublin, No. KE-0254/114/04/2023) was used. Hemolysis and Ca^2+^-activated blood clot formation tests were performed on 50 ± 2 or 100 ± 2 mg samples, respectively (each variant in triplicate for hemolysis and quadruplicate for clot formation), as described elsewhere.^47^ As controls, 0.1% Triton X-100 (positive) and a 50 ± 2 mg piece of high-density polyethylene (HDPE) (negative) in the hemolysis test or a 100 ± 2 mg piece of HDPE (positive) and nonactivated blood (negative) in the clot formation test were used. The concentration of erythrocyte-released hemoglobin (HB) was evaluated on the basis of the Drabkin reagent (Chempur, Poland) reaction and measured at 540 nm using a Synergy H4 Hybrid microplate reader (BioTek). Statistically significant differences between the negative control (hemolysis) or positive control (Ca^2+^-activated blood clot formation) and prostheses samples were considered at *p* < 0.05, according to a one-way ANOVA with post hoc Dunnett’s test (GraphPad Prism 8.0.0 Software, San Diego, CA). A material-activated clot formation test was performed using nonactivated human blood. Modified prostheses samples (50 ± 2 mg) were placed in 0.5 mL Eppendorf tubes containing 0.5 mL of citrated blood and were incubated on a roller mixer RM5-30V (CAT, Germany) at 37 °C and 5 rpm for 2 h. The procedure was followed with subsequent glutaraldehyde fixation (2.5% glutaraldehyde in PBS pH 7.4, 1 h, followed by washing in PBS pH 7.4, twice, and dehydration in subsequent ethanol solutions: 30, 50, 70, 80, and 99.8%, 15 min each step) and SEM evaluation.

### 2.6 Ca^2+^ and PO_4_^3–^ Uptake from SBF

In order to verify the amount of calcium and phosphate ions adsorbed to vascular grafts, 50 ± 2 mg pieces of vascular grafts were immersed in 1 mL of sterile SBF pH 7.42 for 28 days, at 37 °C and 100 rpm (Innova 42, New Brunswick Scientific, Edison, NJ) with weekly exchange of SBF. The concentration of Ca^2+^ and PO_4_^3–^ ions in the collected SBF samples was analyzed using calcium CPC and phosphorus commercial kits (Biomaxima, Poland), according to the manufacturer’s instruction. Absorbance reads were taken using a Genesys 10s UV–vis spectrophotometer (Thermo Fisher Scientific). Afterward, the prostheses samples were air-dried and observed using a scanning electron microscope.

### 2.7 Gentamicin Binding and Release

PLD-coated samples were incubated in a 0.5 mg/mL gentamicin (Merck) solution in a 0.1 M Britton–Robinson buffer with pH 8.5 (50 ± 2 mg of prosthesis fragment per 2 mL of antibiotic solution) for 24 h at 30 °C and 186 rpm in DTS-4 (ELMI, Newbury Park, CA) and then incubated stationary at 4 °C for 24 h. The amount of gentamicin immobilized on the grafts was calculated from the difference in the gentamicin concentration in the drug solution before and after incubation with grafts. The drug concentration was evaluated as described elsewhere,^48^ after phthaldialdehyde (Sigma-Aldrich) derivatization, using a Genesys 10s UV–vis spectrophotometer (Thermo Fisher Scientific). Statistically significant differences between the unmodified prosthesis (P) and the modified prostheses were considered at *p* < 0.05, according to a one-way ANOVA with post hoc Dunnett’s test (GraphPad Prism 8.0.0 Software, San Diego, CA). For drug release, 500 mg of modified prosthesis containing the drug (972.3 μg for P–C–I, 2237.15 μg for P–C–P, 599.4 μg for P–C–H, 323.3 μg for P–C, and 66.1 μg for P) was incubated with 20 mL of sterile PBS with pH 7.4 at 37 °C and 100 rpm (Innova 42, New Brunswick Scientific, Edison, NJ). At selected time points, 0.5 mL samples were collected and replaced by an equal volume of fresh PBS. The amount of released gentamicin was quantified with the phthaldialdehyde method, as described above.

### 2.8 Evaluation of Antibacterial Properties

Before the evaluation of antibacterial properties, the samples were sterilized by the EthO method.

#### 2.8.1 Bacterial Growth Zone Inhibition Test

Sterilized modified prosthesis samples (50 ± 2 mg) with or without immobilized gentamicin were placed in wells (ø = 16 mm) and drilled in Mueller–Hinton (Biomaxima, Poland) agar plates, which were afterward filled with a warm agar medium and left to solidify. Then, 50 μL of a 3 × 10^7^ CFU/mL bacterial inoculate (*S. aureus* ATCC 25 923 or *E. coli* ATCC 25 922) was evenly spread on agar. After 16 h at 37 °C, inhibition zone diameters were measured. The obtained results were compared to inhibition zones measured for Whatman filter paper rings containing 10, 50, and 100 μg of gentamicin. The antibacterial activity test (according to AATCC Test Method 100-2004 “Antibacterial finishes on textile materials: Assessment of...”^49^): 50 ± 2 mg of sterile modified prostheses, both with and without immobilized gentamicin, was placed on sterile inert polypropylene foils, and 30 μL of an *E. coli* suspension (1.5 × 10^4^ CFU/mL) or *S. aureus* suspension (3.0 × 10^3^ CFU/mL) was placed on the sample surface. After 45 min or 3 h of incubation at 37 °C, samples were vigorously washed (to remove the introduced bacteria) with 5 mL of sterile saline, which was further seeded on agar plates (in volume of 50 μL per plate) using an EasySpiral diluter/plater (Interscience, France). As controls, 30 μL of each bacterial suspension diluted in 5 mL of sterile saline was used and treated in a similar way. After 24 h of incubation (at 37 °C), the number of colonies was counted using a Scan 300 colony counter (Interscience, France) to evaluate the number of viable bacteria. The experiment was performed in triplicate. Adhesion test: Prostheses samples (50 ± 2 mg), both with and without immobilized gentamicin, were submerged in 1 mL of 3 × 10^7^ CFU/mL solutions of *S. aureus* or *E. coli* and incubated for 2 h at 37 °C and 50 rpm (Innova 42, New Brunswick Scientific, Edison, NJ). Then, samples were gently washed with sterile saline to remove all nonadherent cells and fixed with glutaraldehyde (Chempur, Poland). In short, prostheses were incubated in 2.5% glutaraldehyde in a PBS pH 7.4 solution for 1 h at RT and then triple-washed with PBS pH 7.4 and dehydrated using an increasing ethanol (POCH, Poland) concentration (as described above), followed by drying at 37 °C. The presence of adhered bacteria was visualized using SEM.

## 3 Results and Discussion

Optimization of the ion-accelerated method for PLD coating of the grafts using 10 mM Tris buffer pH 8.5 as a medium was inspired by seawater ionic composition. In pilot reports,^39,40^ only two main ions present in seawater (Na^+^ and Mg^2+^, in concentrations suggested by the content in seawater) were taken into consideration. In experiments described in this study, Cu^2+^ and SO_4_^2–^ ions were also included in the polymerization medium. The ion concentration in seawater varies substantially, depending on the sea area, depth, season, water pollution, and many other factors. Thus, seawater-related ion concentrations used in this study (472.2 mM for Na^+^, 53.7 mM for Mg^2+^, 10.6 mM for Ca^2+^, 28.2 mM for SO_4_^2–^) constituted the average values of concentration given in literature reports.^41−46^ The content of Cu^2+^, the fifth ion used in this study, is normally low in seawater (1–25 μg/L),^50^ and its higher concentrations are usually associated with anthropogenic sources. This low copper ion concentration was neutral for PLD formation (data not shown). For this reason, 0.5 mM (31,000 μg/L) Cu^2+^ concentration was used in optimization experiments, as it was effectively used elsewhere.^28^ Seawater-related ion concentrations were expected to be the most efficient for PCA polymerization because this process was designed by nature (mussels’ attachment to underwater structures). However, to verify to what extent the seawater-related ion concentration is crucial for PCA polymerization, each ion was also tested at notably higher and lower concentrations (10-fold higher and 10-fold lower values were selected). The efficacy of gentamicin binding to PLD-coated grafts was selected as a criterion, with grafts coated with PLD without any accelerators as the reference.

**Figure 1:**
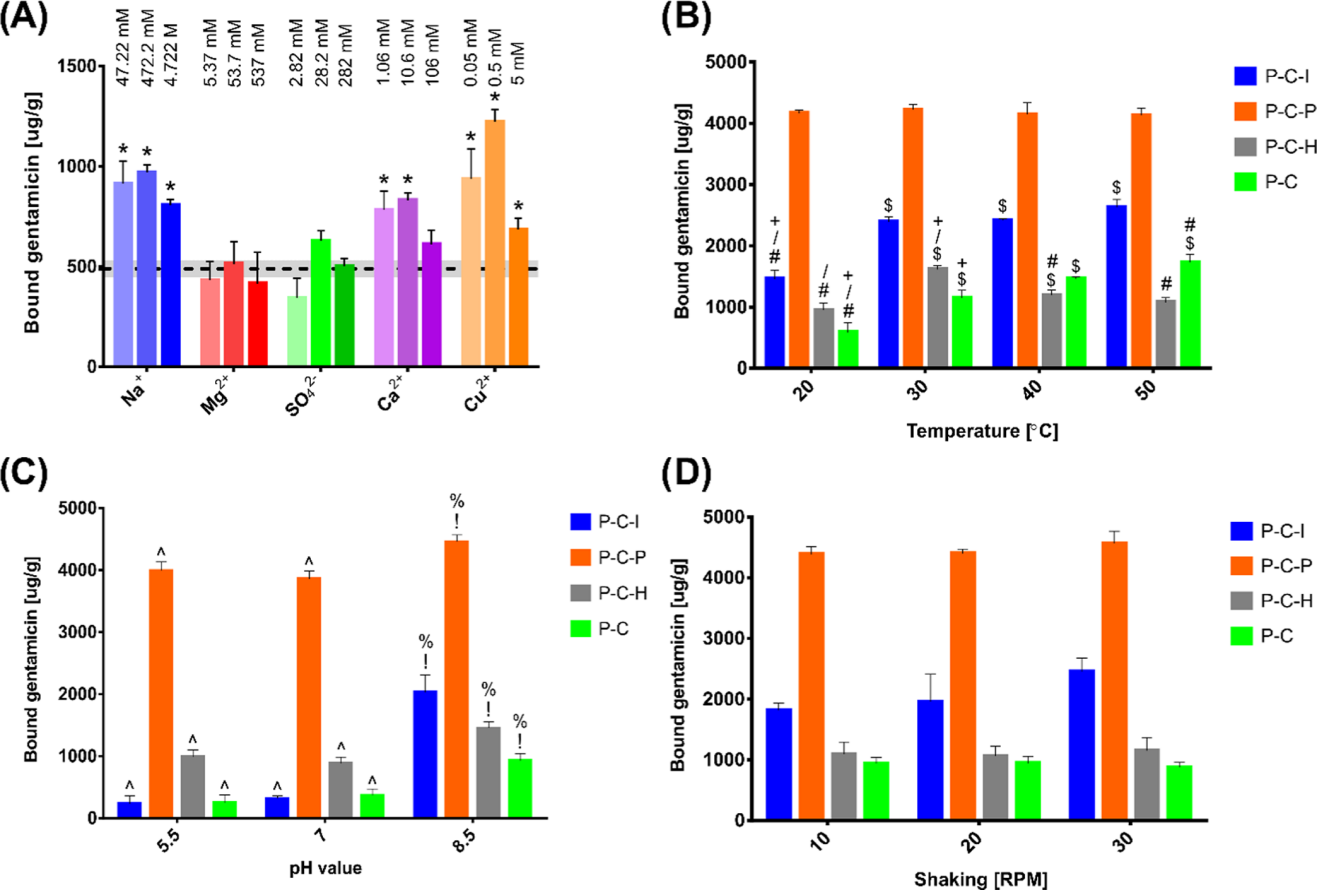
Optimization of parameters of the ion-accelerated poly(l-DOPA) coating method in comparison with the NaIO_4_-accelerated and Cu^2+^/H_2_O_2_-accelerated methods. (A) Effect of concentration of individual ions in the ion-accelerated method; (B) temperature; (C) pH; and (D) shaking speed on l-DOPA polymerization. The dotted line surrounded by the gray zone indicates the mean amount of gentamicin bound to the reference sample (489 μg/g of prosthesis) ± standard deviation. *statistically significant results compared to the reference sample according to one-way ANOVA with post hoc Dunnett’s test (*p* < 0.05); ^$,#,/,+^ or ^%,!,∧^ indicate statistically significant results compared to the samples of the same modification coated at 20, 30, 40, 50 °C or pH 5.5, 7, 8.5, respectively, according to the one-way ANOVA with post hoc Tukey’s test (*p* < 0.05).

First, the concentration of individual ions in 10 mM Tris buffer (pH 8.5) was optimized. According to the obtained results, Mg^2+^ did not significantly affect gentamicin binding in any concentration used (Figure 1A), in comparison with a reference graft (increase by 5% only). Sulfate, calcium, and sodium ions in medium concentrations caused the increase of the drug-binding efficiency by 30, 70, and 98%, respectively. However, for sulfate ions, the increase was not statistically significant. Copper ions were the most effective, also in medium concentrations: it caused the increase of the gentamicin-binding yield by 150% (Figure 1A). Lower and higher concentrations of particular ions were less effective. Strikingly, the selection of ions and their concentration used in pilot studies^39,40^ was close to the optimal choice. Therefore, a combination of all of the tested ions in optimal concentrations (472.2 mM Na^+^, 53.7 mM Mg^2+^, 10.6 mM Ca^2+^, 28.2 mM SO_4_^2–^, 0.5 mM Cu^2+^) was selected for further experiments (named: ion-accelerated method; P–C–I). Further optimization of the PLD-coating process included the choice of the most effective temperature, pH, and shaking speed and was performed for the ion-accelerated modification method (P–C–I), the sodium periodate-accelerated method, and the Cu^2+^/H_2_O_2_-accelerated method, compared to the reference PLD-coating process without any additive (summarization of the modification procedure has been described in Table S1). Temperature, shaking speed, and pH were optimized for P–C–I, P–C–P, P–C–H, and P–C modification procedures within the ranges 20–50 °C, pH 5.5–8.5, and 10–30 rpm. The periodate-accelerated process was temperature-independent, and the Cu^2+^/H_2_O_2_-accelerated one gave the best gentamicin immobilization yield at 30 °C, while for the ion-accelerated and reference process, it was at 50 °C (although small differences were observed within the range 30–50 °C) (Figure 1B). Importantly, cracks and defects appeared in the collagen sealing layer at 50 °C, suggesting the disruption of the collagen sealing layer (data not shown). pH 8.5 was the most beneficial for all methods, although the impact of alkaline pH was more distinct for P–C–I and the reference method in comparison with the other methods (Figure 1C). Shaking speed did not exert any significant impact on the drug-binding efficiency of the resulting grafts, with the exception of P–C–I samples (but the difference was not statistically significant; Figure 1D). Taking into consideration both gentamicin-binding efficiency and collagen layer integrity, for further experiments, the following conditions of PLD coating were selected: 30 °C, pH 8.5, and 30 rpm.

**Figure 2:**
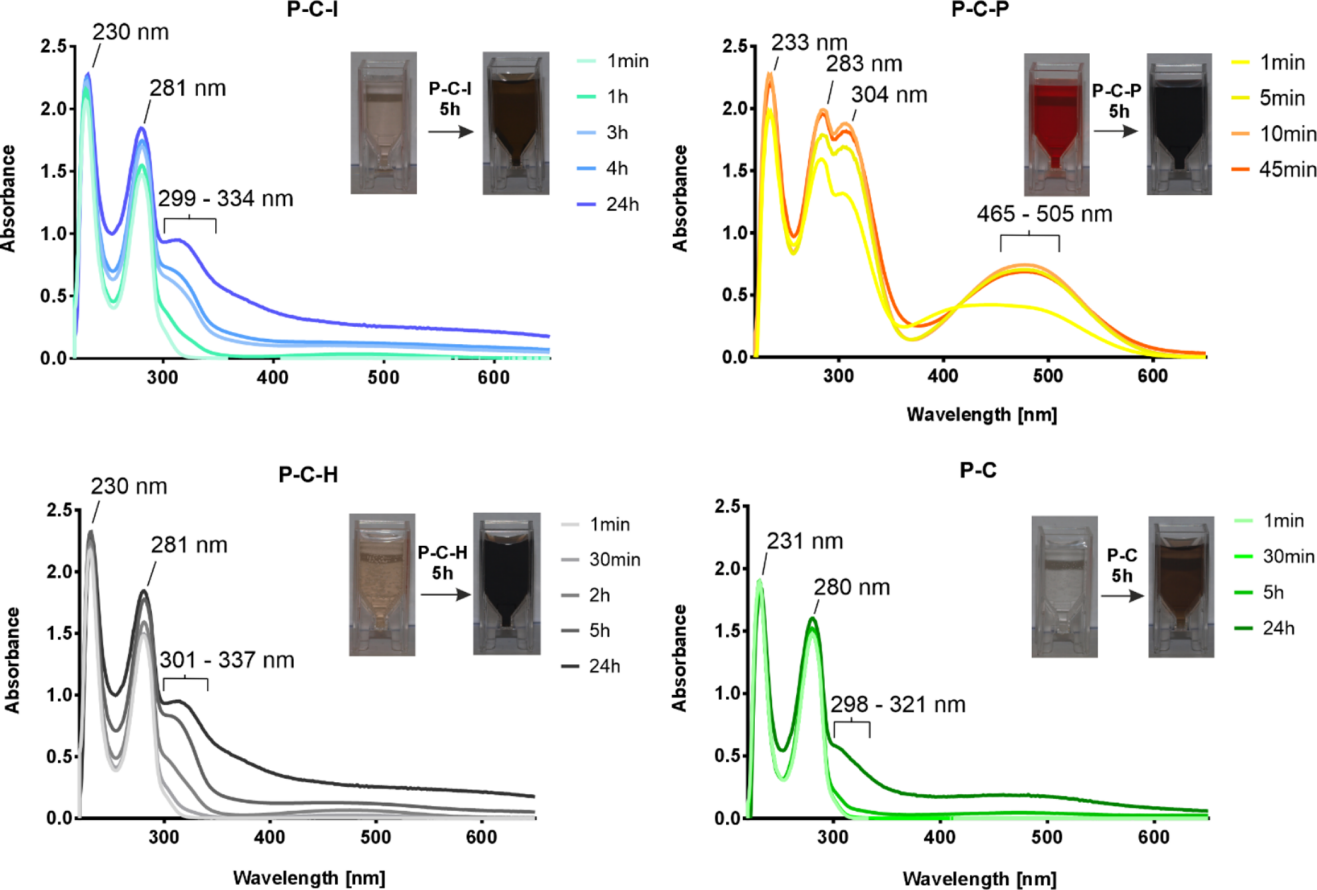
Changes in the absorption spectrum during the PLD polymerization process using different accelerators. In insets: Color change of the reaction mixtures between the 1st minute and the 5th hour of polymerization.

Dynamics of PLD formation was first visualized optically. The color of the reaction mixture observed after 1 min of reaction was light red for the periodate-accelerated method, suggesting the quick appearance of intermediates. At the same moment, a light-pink color was observed for both the Cu^2+^/H_2_O_2_-accelerated and ion-accelerated methods. The reaction mixture for the reference method without accelerators was colorless (Figure 2). After 5 h of reaction, the mixture turned black for the periodate- and Cu^2+^/H_2_O_2_-accelerated methods, dark brown for the ion-accelerated method, and light brown for the reference method (Figure 2). These observations reflect the differences in the speed of PLD polymerization accelerated by the appearance of different agents and intermediators. When sodium periodate was added to the reaction mixture, a flat and broad peak (370–510 nm) appeared within the 1st minute; then, the peak became sharp with a maximum at 480 nm (Figure 2; 5 min). This may indicate the quick appearance of *o*-quinone (390 nm), which turned into aminochrome (304 and 480 nm). During 45 min, the process reached its maximum with no further changes. Significant darkening of the solution was then observed, and the entire process showed high similarity to the sodium periodate-accelerated reaction reported by Ponzio et al.,^28^ suggesting the catecholamine conversion to an indole-type polymer. Ponzio mentioned that when periodate is reduced to iodate, the polymerization of dopamine proceeds with little effectiveness. This remains in agreement with the rate of l-DOPA polymerization observed in this study. For the Cu^2+^/H_2_O_2_-accelerated method, both Cu^2+^ and hydrogen peroxide in the alkaline medium were expected to produce reactive oxygen species, which play a key role in polymerization of catecholamines.^29^ Besides, Cu^2+^ can bind to catechol systems with chelate formation and can induce electron transfer to oxygen to give semiquinone-type species following deprotonation. For the ion-accelerated method, the enhancement of l-DOPA polymerization was also expected because it was reported that some ions other than Cu^2+^ (Na^2+^, Mg^2+^, Ca^2+^) can slowly oxidize dopamine to aminochrome.^51,52^ In the collected spectra (Figure 2) of both ion-accelerated (P–C–I) and Cu^2+^/H_2_O_2_-accelerated (P–C–H) l-DOPA polymerization processes, very slow aminochrome formation detectable at around 304 nm and a flattened band at 480 nm persisting after several hours were observed. This different behavior is similar to that observed by Ponzio et al.^28^ for ammonium peroxodisulfate-mediated catalysis and is probably attributed to the slow kinetics of oxidation, which proceeds via several concurrent pathways. As a consequence, uncyclized dopamine units are likely to be incorporated to a major extent into the growing polymers, with only a low proportion of aminochrome-derived cyclized units. The presence of dopamine-type units in the reaction mixture was proven by the high absorption around 280 nm observed even after 24 h. Thus, nonreacted l-DOPA units were likely to undergo bimolecular incorporation into the growing polymer. For the control spectrum (P–C), evolution of the aminochrome intermediate (304 nm) was much slower than for the other three methods, supporting the conclusion that all accelerators of l-DOPA polymerization (combination of ions, sodium periodate, and Cu^2+^/H_2_O_2_) efficiently increased the process of PLD formation. Ion-accelerated (P–C–I) and Cu^2+^/H_2_O_2_-accelerated methods showed the most similarities.

**Figure 3:**
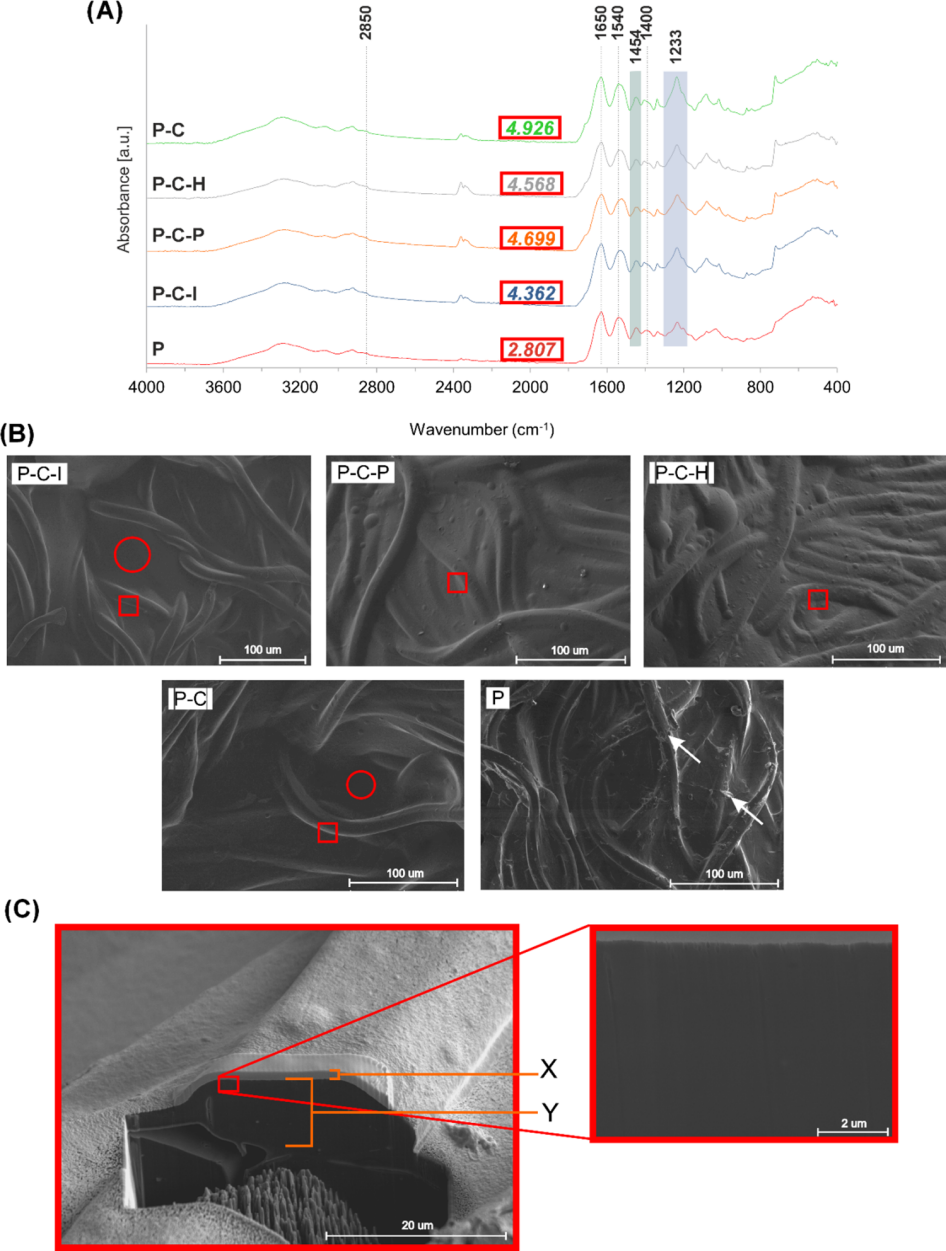
Characterization of the prosthesis with the PLD interface. (A) FTIR spectra of the composites and their compounds with ratios of 1233 to 1454 cm^-1^ intensity for each spectrum (in red frames). (B) SEM pictures of the modified grafts. Red circles show areas between the polyester fibers filled with collagen; white arrows point to irregularities on the graft surface; and red rectangles indicate the edges of the fibers. (C) FIB-SEM image of the cross section of the prosthesis coated with PLD (P–C–I; magnification 2500×). The inset (marked in the red rectangle) shows the magnified fragment of the upper surface of the PLD-coated prosthesis (depth of approximately 4 μm) with the layer of sputtered Au (X) and the layer of collagen + PLD coating (Y).

FTIR spectra of vascular prostheses coated with PLD using different stimulants did not reveal spectacular differences. The presence of PLD was detected in modified spectra. The 1410 cm^–1^ band is characteristic of the C–O–H bond in catechol groups, as shown in the study of Zhu et al. concerning the synthesis of collagen-polydopamine hydrogels.^53^ In the tested prosthesis, the 1400 cm^–1^ band present in the uncoated prosthesis shifted to 1408 cm^–1^, suggesting the presence of catechol groups in all coated grafts. Similarly, the 1540 cm^–1^ band observed in the uncoated prosthesis shifted in spectra of modified grafts to 1532 cm^–1^ for P–C–I, to 1533 cm^–1^ for P–C–H, and to 1523 cm^–1^ for P–C–P, with no shift for the P–C prosthesis. This shift is likely to be caused by the presence of PLD, which shows an absorption at 1512 cm^–1^.^54^ The small increase of absorbance at 2850 cm^–1^, characteristic of the catechol −OH group in polydopamine,^55^ was also observed in the spectra of all coated prostheses in comparison with the uncoated one (Figure 3A).

The main bands indicating the presence of collagen on modified grafts (amide I at 1650 cm^–1^ and amide II at 1540 cm^–1^) were clearly present in all of the spectra. This proved that the tested methods of PLD deposition allowed the protein to remain on the grafts. The position of amide III (∼1310 to ∼1175 cm^–1^), a sensitive marker of changes in the secondary protein structure,^56^ remained unchanged in the spectra of all coated biomaterials, suggesting the existence of a collagen helical structure. However, the ratios between amide III (1233 cm^–1^) and 1454 cm^–1^ bands were calculated, which should be close to 1 in native collagen.^57^ For uncoated prosthesis, the 1233/1454 cm^–1^ ratio was 2.807, suggesting the possible alteration in the secondary structure of collagen during the prosthesis fabrication process (the prosthesis was sealed by dip-coating in a collagen solution, followed by drying). For all PLD-coated grafts, the 1233/1454 cm^–1^ ratio increased to 4.362–4.926, suggesting a more distinct change in the secondary structure of collagen during the coating process (Figure 3A). The mechanism of this change rather excludes collagen degradation because Plepis et al.^58^ in their study showed the 1233/1454 cm^–1^ ratio equal to 0.59 for partially degraded collagen (gelatin) in comparison with the 1233/1454 cm^–1^ ratio of ∼1.0 for native collagen of a triple helix preserved structure. The most probable explanation is the reaction between the thiol and amino groups of collagen and catechol moieties of PLD. These interactions likely strengthen the integration of the PLD interface with the collagen matrix.

SEM observations of modified graft surfaces provided some suggestions concerning PLD-layer deposition on grafts. The control uncoated graft (P) showed the presence of weaves of polyester fibers with spaces filled with a layer of collagen (Figure 3B). Irregular shreds occur on the polyester fibers, suggesting the presence of collagen also on the surface of the sharply outlined fibers. The surface of the PLD-coated prostheses was smoother and no longer contained irregular shreds, suggesting that they were covered with a thin layer of PLD. Also, the edges of polyester fibers became less sharp as if they were covered with a layer of dust. This effect is especially visible for the P–C–P and P–C–H prostheses, which may suggest the presence of a thicker layer of PLD deposited on the surface of the prostheses in comparison with P–C–I and in particular with P–C (Figure 3B). These observations are in agreement with the color change rate observed in the reaction mixture (Figure 2).

The FIB/SEM technique was used to prepare the cross sections of the coated grafts and to verify the thickness and tightness of adhesive PLD layers. Figure 3C shows the cross section of the P–C–I graft, which is representative of all studied prostheses. The evaluation of the thickness of the PLD layer failed because all polymers (PLD, collagen, and polyester) showed the same contrast in the image. However, no delamination in cross section within the upper surface region (marked within a red rectangle) of an approximate depth of 4000 nm (Figure 3C, inset) was observed. As PCA layers reported in the literature are usually maximum several dozen nanometers thick (≤45 nm^59^), lack of delamination within this area supports the hypothesis that PLD deposition on collagen-sealed grafts is tight and stable.

**Figure 4:**
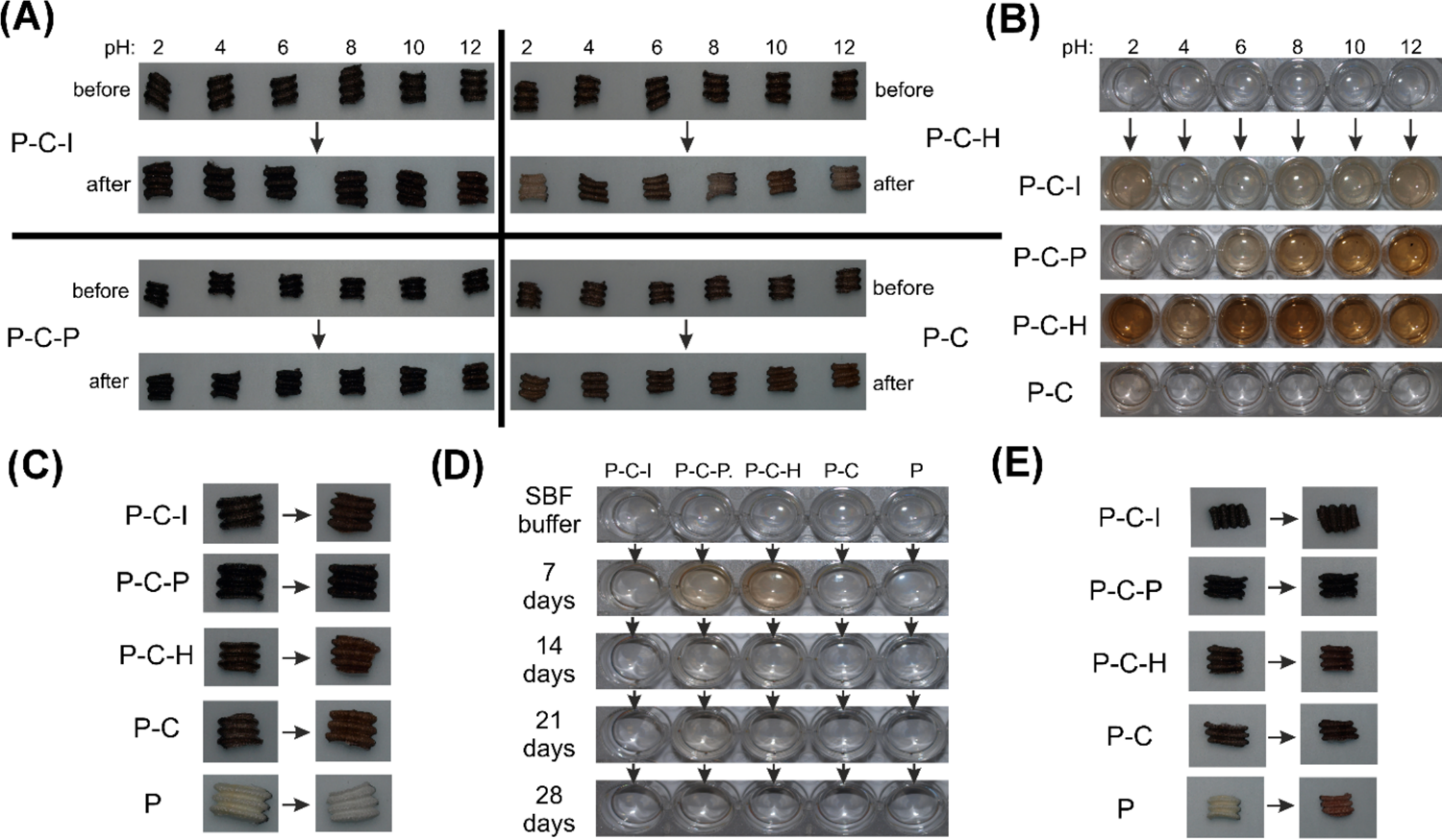
Stability of coated prostheses in different media. (A) Images of prostheses before and after incubation in Britton–Robinson buffer pH 2–12 after 7 days of incubation; (B) color change of Britton–Robinson buffer pH 2–12 after 7 days of incubation with modified prostheses; (C) images of prostheses before and after incubation in SBF; (D) color change of SBF incubated with prostheses during the test; and (E) images of prostheses after 3 days of incubation in human blood.

The main purpose of modifying vascular prostheses with PLD is to conjugate them with an antibiotic and thus protect them against bacterial infection. PLD coatings should therefore be stable both in tissue fluids (for the outer side of the prosthesis) and in blood (for the prosthesis lumen). For this reason, the modified prostheses were incubated in SBF H 7.42 for 28 days, with the medium changed weekly. All prostheses remained black within the entire period of the experiment, with a slight increase of their brightness indicating delicate instability of the PLD coating (Figures 4C and S1B). No significant differences between all types of modified grafts were observed until the SBF color was measured (Figures 4D and S1A). Grafts P–C and P–C–I affected the SBF color only during the first exchange; then, SBF remained colorless, indicating further stability of the PLD coating. However, samples P–C–P and P–C–H showed higher coating instability. SBF was tinted due to the release of PLD during the entire period of incubation, although this phenomenon was the most intense during the first test period. As PLD coating serves as the interface/platform for antibiotic binding, the instability of the PLD layer in P–C–P and P–C–H samples may suggest that despite the high drug-binding efficacy they may release the immobilized drug quicker in comparison with the other tested grafts. This observation may suggest an advantage of the ion-accelerated method over other accelerated methods of PLD deposition.

Incubation of PLD-coated grafts in human blood showed a high stability of the PLD layer. No changes in color were observed for P–C–I, P–C–P, and P–C samples. A similar stability of PLD layers in human blood was also observed in another report, concerning polycatecholamine formation on a polysaccharide hydrogel.^47^ Slight brightening was noted only for P–C–H, suggesting the lowest stability of the PLD layer in blood (Figures 4E and S1E). It should be noted that the color change of the studied prostheses before and after incubation in blood can originate both from the dissolution of the PLD layer and from the deposition of fibrin clot and erythrocytes on the surface of the samples. The second phenomenon was clearly observed for the control uncoated prosthesis (P), which exhibited significant darkening due to red blood cell attachment (Figures 4E and S1E).

The PLD-coating stability of the studied prostheses was also tested in buffers with different pH values for 7 days, which allowed us to note the differences between the tested modification methods. The color of both the grafts and postincubation buffers was investigated. The prostheses themselves remained dark after incubation in the buffers. Notable brightening was observed only for the P–C–H graft incubated at pH 2 (which is out of the physiological pH range) (Figures 4A and S1D). The color of postincubation buffers revealed more details. Reference P–C samples were the most stable (at all pH values), with very slight instability detected only at pH 2. P–C–I samples were also relatively stable (slight instability at pH 2 and 10–12). P–C–P samples were unstable within the pH range of pH 6–12, whereas P–C–H samples showed instability within the entire pH range (Figures 4B and S1C). Overall, PLD coating prepared with the ion-accelerated method showed the highest stability among all accelerator-supported methods.

**Figure 5:**
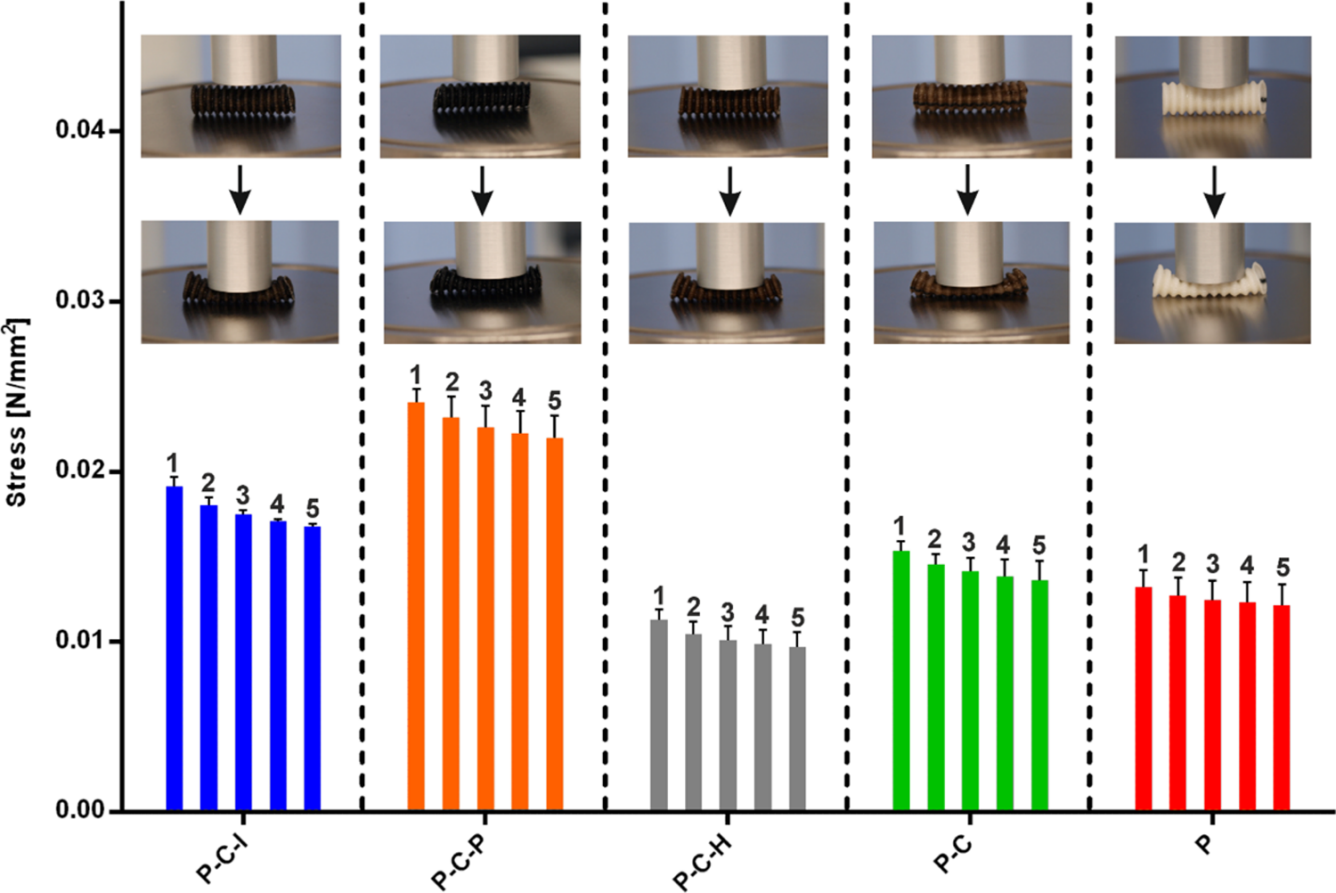
Stress detected during 50% graft compression in a repeated cycle. Numbers 1–5 indicate the cycle of compression (*n* = 3).

Mechanical properties are important features of vascular prostheses, which must resist repeatable expansion–release cycles *in vivo*. Thus, to evaluate the mechanical resistance of PLD-coated prostheses, the samples were subjected to 50% compression repeated 5 times. P–C–P samples showed the highest detected stress during compression, followed by P–C–I, P–C, P, and P–C–H. The obtained values show a trend indicating the relationship between the compressive strength and the intensity of PLD-coating formation: the highest for P–C–P, and the lowest for P–C. P–C–P, P–C–I, and P–C showed 181, 141, and 114% stress of the control samples during the compressions (Figure 5). These findings are in agreement with literature data, which suggest the improvement of mechanical properties of biomaterials under polydopamine addition.^60^ Surprisingly, the P–C–H sample, which was expected to show a mechanical parameter similar to that of the P–C–P sample, broke out of this trend: the stress was the lowest for this graft (Figure 5). The drop in stress of this sample may result both from the relative instability of PLD coating and the degradation of polyester fibers caused by free radicals.^61,62^ As reported, hydroxyl free radicals could be generated from H_2_O_2_ in the presence of copper ions.^63^ For all samples, stress decreases within the cycle, suggesting a slight dislocation of polyester fibers during repeated loads.

**Figure 6:**
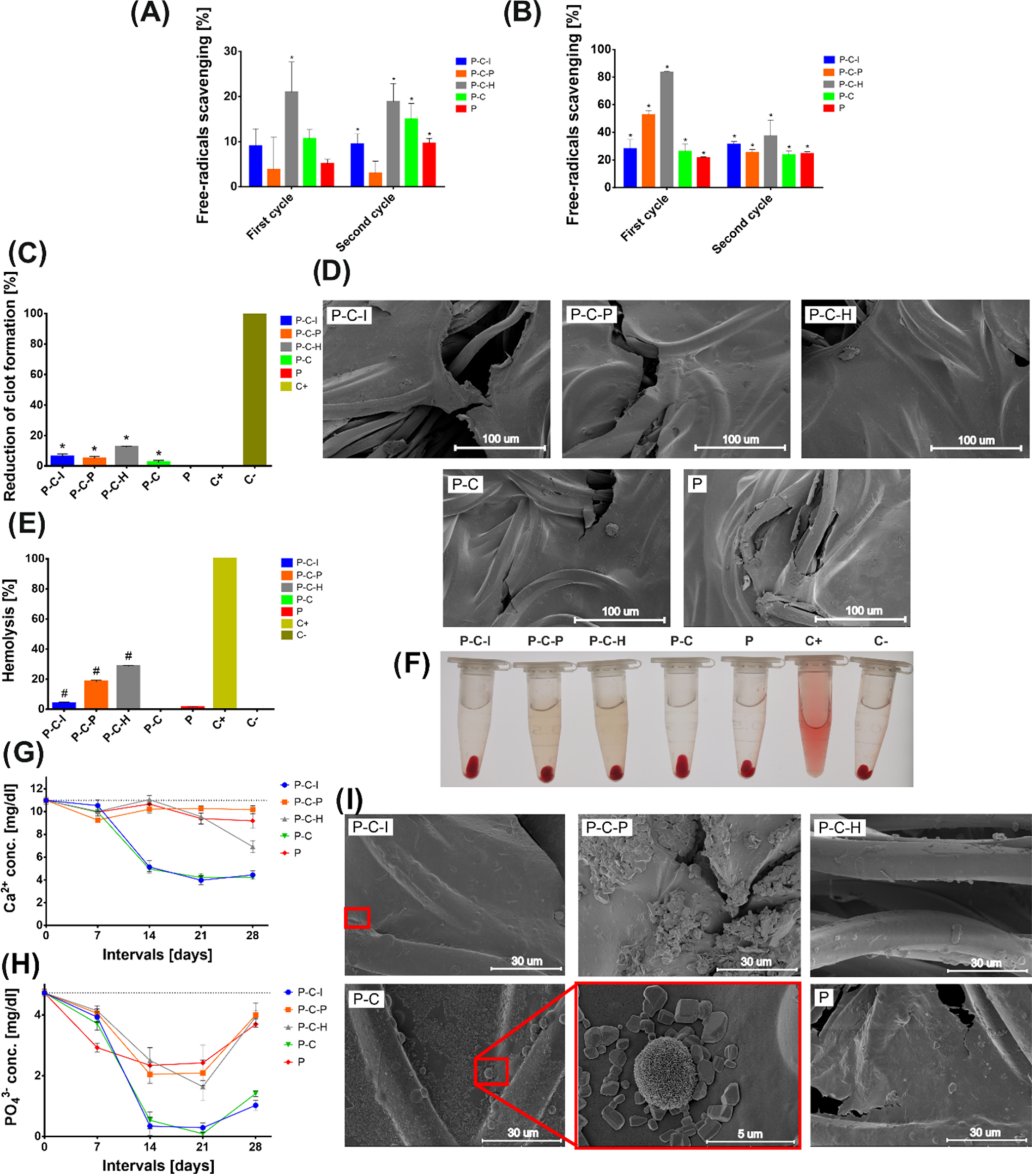
Antioxidant properties of the modified prostheses in the (A) DPPH assay and (B) ABTS assay (*statistically significant results compared to the control without prostheses according to one-way ANOVA with post hoc Dunnett’s test (*p* < 0.05)). (C) Clot formation in Ca^2+^-activated blood (*statistically significant results compared to the positive control according to one-way ANOVA with post hoc Dunnett’s test (*p* < 0.05)). (D) Clot formation in material-activated blood by SEM visualization. (E, F) Hemolytic properties of modified vascular grafts (^#^statistically significant results compared to the negative control according to one-way ANOVA with post hoc Dunnett’s test (*p* < 0.05)). Absorption profiles of (G) Ca^2+^ and (H) PO_4_^3–^ by modified prostheses in SBF. The dotted line indicates the ion levels in SBF. (I) SEM images of the prosthesis substrates incubated in SBF; magnification 1.500×. Red rectangles indicate the hydroxyapatite crystals’ deposits.

PCAs are known for their antioxidant properties.^64^ This feature can be beneficial for vascular prostheses because free radicals in blood increase the risk of arteriosclerotic plaque formation.^65^ Therefore, the antioxidant properties of PLD-coated grafts were evaluated using DPPH and ABTS assays based on scavenging of the chemical radicals by antioxidants. According to ABTS assay results, P–C–I and P–C grafts showed a similar antioxidant activity as the bare P graft. In turn, the P–C–H sample exhibited the highest antioxidant activity, followed by the P–C–P sample (Figure 6A,B). Ponzio suggested that when dopamine polymerization is accelerated by sodium periodate, small quantities of iodine are incorporated into the synthesized material.^28^ Iodine (in the form of I^–^) was reported to show antioxidant properties;^66^ thus, some PLD-incorporated iodine may explain the enhanced antioxidant properties of P–C–P samples. However, in the second assay cycle, the antioxidant activity of P–C–H and P–C–P samples was significantly reduced (Figure 6A,B). This suggests that the antioxidant activity of the PLD-coated prosthesis is unstable and should not be considered as a notably beneficial feature of PLD-modified vascular grafts.

The results in the DPPH assay were less reliable than the ABTS one due to very high SD values, but also in this assay, the P–C–P sample showed the highest initial free radical scavenger activity (Figure 6A,B).

An extremely important feature of vascular prostheses is human blood safety upon contact with the biomaterial. According to the ASTM F756 standard (“Standard Practice for Assessment of Hemolytic Properties of Materials”),^67^ the hemolytic index (HI) should be 0–2 for nonhemolytic materials, 2–5 for slightly hemolytic ones, and >5 for hemolytic ones. Test of blood hemolysis upon contact with PLD-coated grafts suggested that P–C–H and P–C–P prostheses were hemolytic as they caused almost 28% (HI = 28) and 18% (HI = 18) blood hemolysis compared to the negative control, respectively. P–C–I modification only slightly increased the hemolysis rate (HI = 3.6). As for the classic reference method of modification (P–C), it did not change this parameter, which is similar to the pristine P graft (Figure 6E,F). Test of clot formation based on measurement of hemoglobin released from erythrocytes nonentrapped within the formed clot showed that all PLD-coated prostheses reduce the clot formation, in particular P–C–H ones (Figure 6C). However, this effect may be masked by the observed hemolysis (Figure 6E,F). For this reason, a material-activated clot formation test was also performed with subsequent SEM observation. All blood-incubated sample prostheses showed the absence of clot and erythrocytes on the surfaces, confirming the lack of thrombosis-promoting activity of PLD-coated collagen-sealed prostheses (Figure 6D). Although PCAs are used in synthesis of some biomaterials to enhance their hemostatic properties,^68^ this effect was fortunately not detected for coated vascular grafts.

PCAs are known as biomineralization stimulants because surface-anchored catecholamine moieties enrich the interface with Ca^2+^, facilitating the formation of hydroxyapatite crystals.^69^ Recently, it was found that nanohydroxyapatite may accelerate *in vivo* vascular calcification.^70^ This may increase the risk of arteriosclerosis plaque formation, which poses a threat to the appropriate functioning of the implanted vascular prostheses. Therefore, the PLD-coated grafts were immersed in SBF for 28 days to verify the possible adsorption of calcium and phosphate ions by the grafts (indirect test) and formation of nanohydroxyapatite on their surface (direct test). Absorption of Ca^2+^ and PO_4_^3–^ by prostheses was detected but only for some of the tested modifications. P–C–I and P–C showed a similar trend: partial (up to 60%) calcium ion absorption and almost total phosphate ion adsorption (up to 97%) (Figure 6G,H). Moreover, this trend seemed to increase starting from the second immersion in SBF, suggesting that the preconditioning of the grafts in SBF played a significant role in ion adsorption. Inversely, P–C–P and P–C–H did not show any tendency to absorb calcium ions (with the exception of P–C–H at the end of the test) and a reduced ability to absorb phosphate ions. This behavior was similar for unmodified prosthesis P (Figure 6G,H). To verify these observations, the samples were subjected to SEM observations (Figure 6I). Although different crystals were found on all samples, characteristic flower-like deposits of apatite were observed exclusively on P–C–I and P–C (Figure 6I, in red rectangles), which correlated with the ion adsorption observed for these particular modifications (Figure 6G,H). Deposits of apatite were more abundantly present and more uniformly distributed on the surface of P–C grafts, as shown on images with a lower magnification (Figure S2).

**Figure 7:**
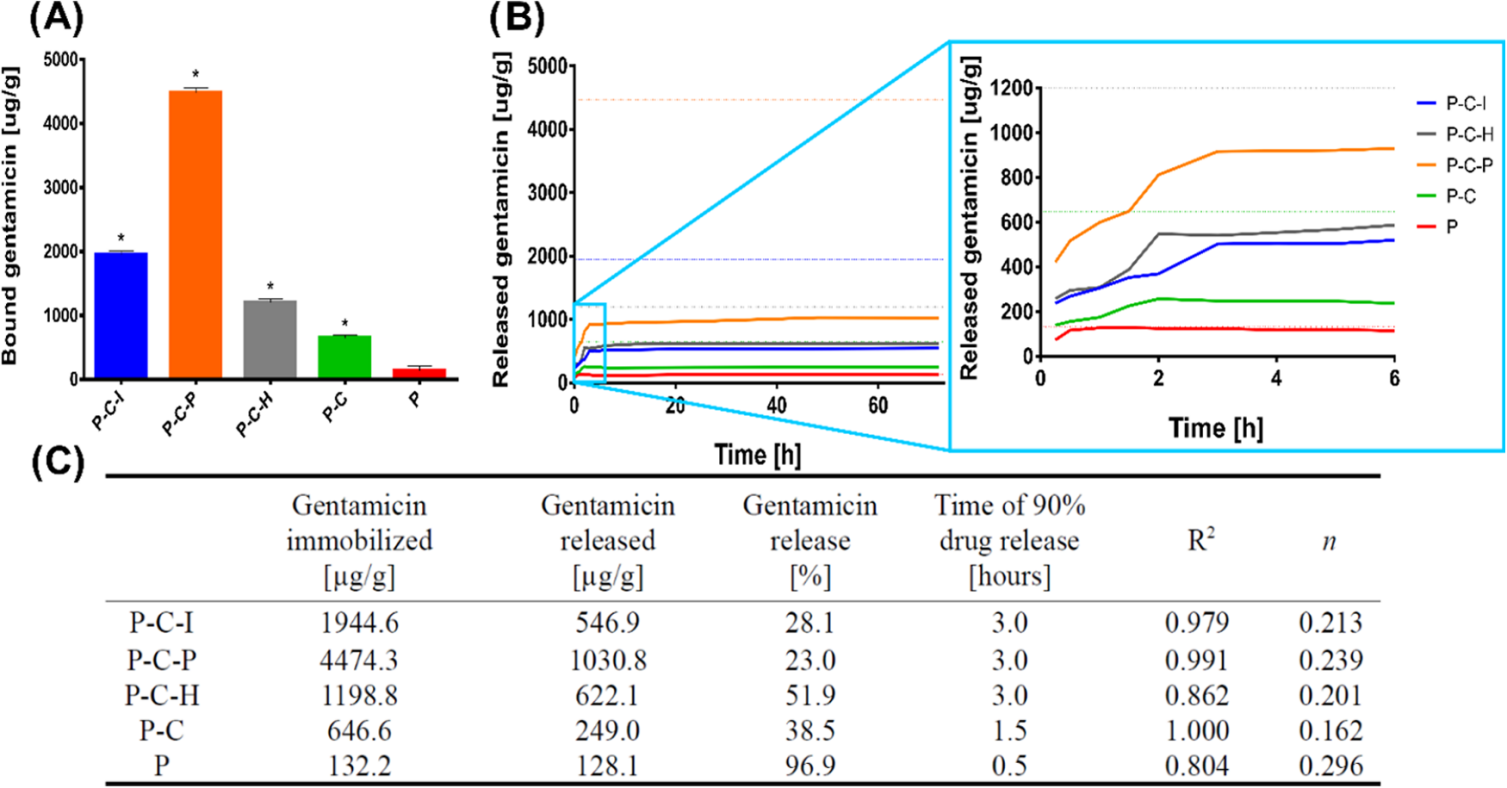
(A) Gentamicin (G) immobilization yield and (B) drug release profile. Dotted lines: initial drug amount for particular samples (per gram) (*statistically significant results compared to the unmodified prosthesis (P) according to one-way ANOVA with post hoc Dunnett’s test; *p* < 0.05). (C) Drug release parameters; *R*^2^, coefficient of determination; *n*, drug release exponent (Korsmeyer–Peppas).

The most important feature of PLD-modified vascular prostheses, from the point of view of this study, is the ability to bind the antibiotic through the PLD coating and the resulting antibacterial properties. Gentamicin was used as a model drug, although it should be noted that the properties of PCAs allow the immobilization of any drug containing a free amino or thiol group in the molecule. Therefore, gentamicin was immobilized to all tested PLD-coated grafts in optimized conditions to check for drug immobilization yield, drug release properties, and the antibacterial activity of the resulting active prostheses. As in the optimization test (Figure 1), the most effective in drug binding was the P–C–P sample (4474 μg/g graft), followed by the P–C–I sample (1944 μg/g graft), the P–C–H sample (1199 μg/g graft), and the P–C sample (647 μg/g graft). All PLD-coated grafts were able to bind significantly more gentamicin than the uncoated prosthesis (P) (Figure 7A,C). Time of drug release was quite short: 0.5–3 h. However, while the P sample released 97% of the drug, all PLD-coated grafts released only 23–52% of the drug; the remaining part was stably bound to the samples (via catechol domains of PLD). It confirmed once again the previous findings that suggested that PCA coatings bind active molecules with free amino groups via both stable and unstable modes.^39,40,47^ Among them, P–C–H released the maximum amount of drug (52%), which was probably related to instability of the PLD coating, as suggested in earlier tests (Figures 4 and S1). The Korsmeyer–Peppas model suggested a pore-dependent (Fickian) type of drug release, although the applied model did not fit optimally for P–C–H and P samples (Figure 7C). The obtained results suggest that the periodate- and ion-accelerated PLD coatings of polyester vascular prosthesis allow for the most effective gentamicin immobilization.

The antibacterial activity of PLD-coated grafts was verified against *S. aureus* (Gram-positive) and *E. coli* (Gram-negative) strains. First, the measurement of bacterial growth inhibition zones around the graft samples was performed. Bacterial growth inhibition zones correlate with the amount of gentamicin immobilized on prostheses. For both species of used bacteria, all of the grafts modified with PLD proved to be more efficient than the P graft. Activities of P–C–I, P–C–H, and P–C–P samples for both bacteria corresponded to the release of 10–50 μg of gentamicin, while for P–C and P, it corresponded to less than 10 μg of gentamicin. For gentamicin-free (**G–**) samples, no antibacterial activity was observed (suggesting that PCA, which is known for its antibacterial properties, was not released from the grafts) (Table S2).

**Figure 8:**
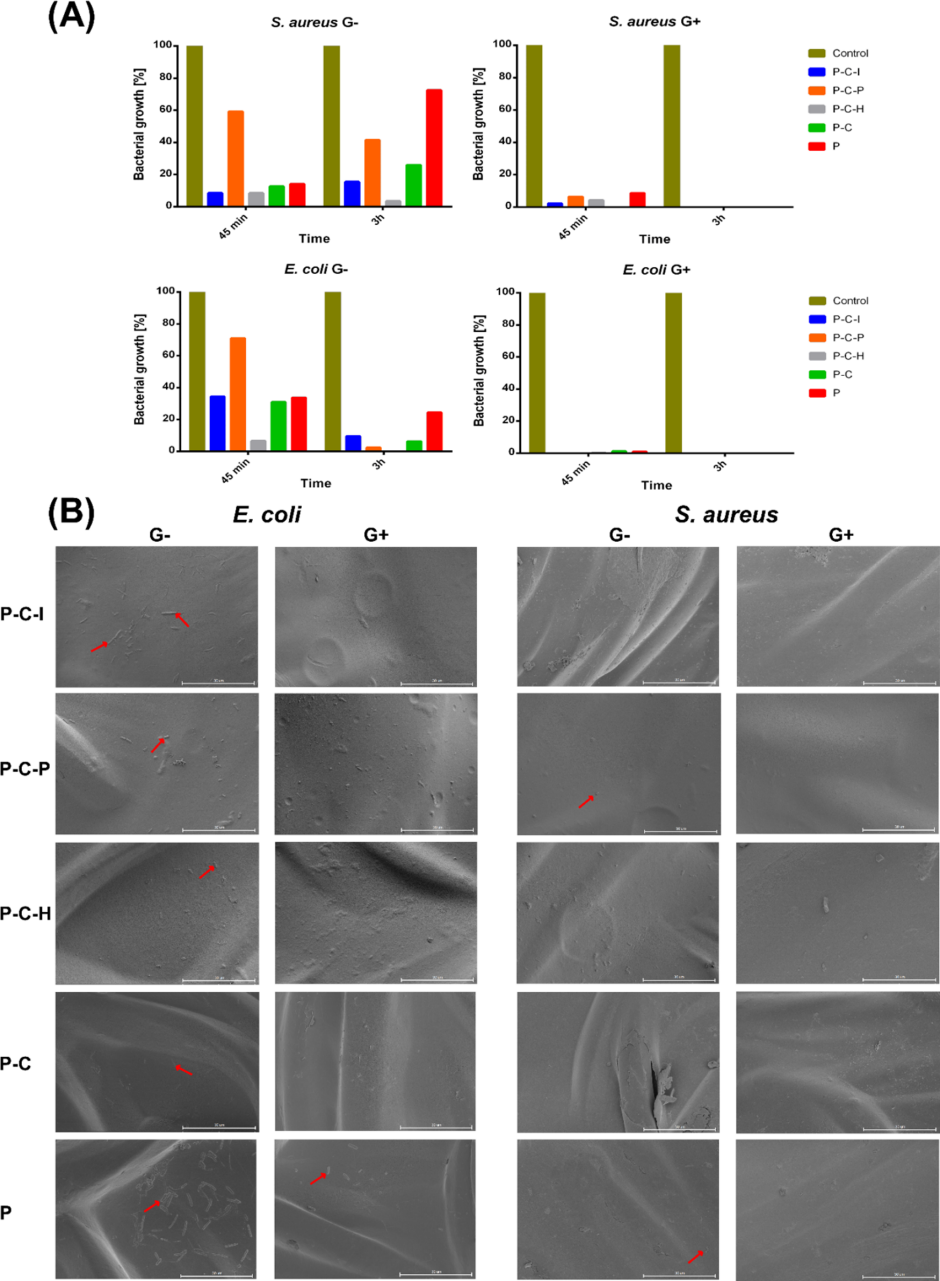
Antibacterial activity of PLD-coated vascular grafts (G, without gentamicin; G+, with gentamicin). (A) Antibacterial activity according to the AATCC Test Method 100-2004.^49^ (B) SEM pictures of bacterial adhesion on modified prostheses. Red arrows indicate the presence of bacterial cells.

Evaluation of the antibacterial activity according to the AATCC Test Method 100–2004^49^ showed high efficacy of all applied methods of PLD coating. Gentamicin-bound grafts killed all bacteria after only 3 h of contact (and the majority (more than 90%) of the bacteria during 45 min) (Figure 8A). However, PLD-coated grafts without a bound drug (**G–**) also showed notable antibacterial activity, although much lower than **G+** grafts. It was expected due to the widely reported antibacterial activity of PCAs.^71,72^ Evidently, P–C–H grafts were the most efficient in bacteria elimination (perhaps due to relative instability of the coating), while P–C–P samples were less effective. The last observation was surprising because P–C–P grafts were expected to reveal a higher pathogen-killing force on the basis of their PLD-formation rate and SEM-concluded thickness. For all samples, the *E. coli*-killing efficacy increased with time (was higher after 3 h than after 45 min). However, for *S. aureus*, this trend was observed only for P–C–P and P–C–H samples, contrary to the remaining samples, suggesting a lower toxicity of the latter to this particular bacterial strain (Figure 8A). In general, the experiment confirmed the notable antibacterial activity of PCAs,^72,73^ both as a coating and as an interface for immobilization of antibacterial drugs.

Considering the adhesion of bacteria, only singular *S. aureus* cells were found attached to pristine grafts, with no attachment observed for all PLD-coated prostheses. Apparently, the PLD layer itself protected the grafts against bacterial adhesion because no difference was noted for samples with and without gentamicin. In turn, *E. coli* adhered to all types of grafts without an antibiotic, although the most abundantly to pristine prosthesis. This suggested that the PLD layer protected the collagen-sealed grafts against *E. coli* adhesion but less efficiently than against *S. aureus*. Further graft coupling with gentamicin completely eliminated the adhesion of *E. coli*; only pristine grafts soaked in antibiotics showed some bacterial adherence to the surface. Thus, it was confirmed that the PLD coating may significantly protect collagen-sealed prostheses against bacterial adhesion, minimizing the risk of bacterial biofilm formation (Figure 8B).

To summarize, evaluation of properties of collagen-sealed vascular grafts coated with PLD, with the addition of an optimized combination of ions, sodium periodate, or Cu^2+^/H_2_O_2_, revealed some differences and similarities between the matrices, highlighting the advantages and disadvantages of different accelerators (Table 1).

**Table 1:** Advantages and Disadvantages of Used Accelerators^a^

	P–C–I	P–C–P	P–C–H	P–C
coating stability	+++	+	–	+++
gentamicin binding	++	+++	++	+
mineralization	+	–	–	+
mechanical strength	++	+++	–	+
antioxidative properties^b^	++	++	+++	++
hemolysis	+	++	+++	–
reduction of blood clotting	+	+	++	+
reduction of bacterial adhesion	++	+++	+++	++
antibacterial activity	++	+	+++	++

a “+” low, “++” medium, “+++” high.

b Short-term activity.

Namely, it seems that relatively aggressive oxidants (sodium periodate and Cu^2+^/H_2_O_2_) allowed the immobilization of more antibiotics on the resulting PLD layer than other accelerators but simultaneously destabilized the PLD coating and caused significant hemolysis. Sodium periodate-accelerated PLD coating enabled gentamicin to bind with the highest yield and showed the highest drug release, whereas the amount of drug bound by P–C–H was relatively low (lower than that for P–C–I), probably due to the reduced stability of the Cu^2+^/H_2_O_2_-accelerated PLD layer. Also, the overall antibacterial activity of the grafts coated with all tested accelerators was relatively similar, although the P–C–P graft was surprisingly less antibacterial-active. The sodium periodate-accelerated method increased the coated grafts the most, while the use of Cu^2+^/H_2_O_2_ resulted in the reduction of mechanical resistance.

However, all grafts showed a similar stability in blood and SBF and a lack of significant impact on blood clot formation, which is very important for vascular prostheses.

The aim of this study was to investigate the effect of selected accelerators on the properties of vascular prostheses. However, it cannot be denied that a surprisingly positive outcome of the functionality of prostheses modified without the use of accelerators (P–C) was observed. This reference variant of the modified prosthesis, despite a clearly lower antibiotic binding capacity and compressive strength, was distinguished by the highest coating stability in various pH levels, the lowest hemolytic index, and a relatively high antibacterial activity (no adhesion of *S. aureus* cells to the surface). It should therefore be emphasized that the reference method may prove to be relatively better than accelerated methods in certain applications where the main requirement is high stability and blood compatibility. However, the selection of an appropriate method for modifying a specific biomaterial should be carefully made based on a wide range of tests.

## 4 Conclusions

As expected, numerous differences were found between the properties of collagen-sealed vascular grafts modified with a PLD layer using different accelerators of PCA formation. The use of quite aggressive oxidants, such as sodium periodate and hydrogen peroxide, may increase the rate of formation of the PLD layer and increase its thickness but at the same time increase the hemolytic properties of the prostheses. Moreover, hydrogen peroxide may reduce the mechanical strength of the prosthesis and destabilize the PLD layer, which may consequently bind less antibiotic than expected. It therefore seems obvious that the appropriate selection of accelerator of PCA formation is extremely important for a particular medical device and it should be based on careful analysis of a wide range of biological properties. As a consequence, a medical device with optimal properties and safety may be obtained.

Moreover, this study suggests that seawater-inspired ion combination merits attention in the design of functionalized medical devices as a novel accelerator of polycatecholamine formation. This accelerator appears to be both relatively safe and effective in selected medical applications.

## Supporting Information Available

Optimization of parameters of the ion-accelerated poly(l-DOPA) coating method in comparison with NaIO_4_-accelerated and Cu^2+^/H_2_O_2_-accelerated methods; influence of addition of single ions, different values of temperature, pH, and shaking; changes of the absorption spectrum during PLD polymerization; FTIR spectra of the composites and their compounds with ratios of 1233–1454 cm^–1^ intensity for each spectrum; SEM pictures of modified grafts; FIB-SEM image of the cross section of coated prosthesis; stability of the coated prostheses in Britton–Robinson buffer pH 2–12 and SBF after 7 days of incubation and color change of the used buffers; images of prostheses after 3 days of incubation in human blood; stress detected during 50% graft compression in repeated cycles; antioxidant properties of the modified prostheses; clot formation in Ca^2+^-activated or in material-activated blood: SEM visualization; hemolytic properties of modified vascular grafts; absorption profiles of Ca^2+^ and PO_4_^3–^ by the modified prostheses in SBF; SEM images of the prosthesis substrates incubated in SBF; gentamicin immobilization yield and drug release profile; drug release parameters; antibacterial activity of PLD-coated vascular grafts with or without gentamicin; and SEM pictures of bacterial adhesion on modified prostheses (PDF)
