# Supplementary material for: Comparison of the Impact of NaIO4-Accelerated, Cu2+/H2O2-Accelerated, and Novel Ion-Accelerated Methods of Poly(l-DOPA) Coating on Collagen-Sealed Vascular Prostheses: Strengths and Weaknesses
Source: ACS Appl Mater Interfaces. 2024 Jul 24;16(31):40515–30. doi: 10.1021/acsami.4c05979 (PMC11310904; doi:10.1021/acsami.4c05979)
Supplement: Supplementary file 1 — am4c05979_si_001.pdf [file am4c05979_si_001.pdf]

Supporting Information

Comparison of impact of $NaIO_4$-accelerated, Cu^{2+}/H_2O_2-accelerated and novel ions-accelerated method of poly(L-DOPA) coating on collagen-sealed vascular prostheses - strengths and weaknesses

Michał Fornal[1], Agnieszka Krawczyńska[2], Anna Belcarz[1]*

[1]Chair and Department of Biochemistry and Biotechnology, Medical University of Lublin, Chodźki 1, 20-093 Lublin, Poland

[2]Faculty of Materials Science and Engineering, Warsaw University of Technology, 141 Wołoska, 02-507 Warsaw, Poland

Corresponding Author

 Corresponding author. E-mail address: anna.belcarz@umlub.pl (A. Belcarz).

Table S1. Prosthesis coating description.

Code	Description of Prosthesis	Coating procedure
P-C-I	Coated by PLD with Ions	1. Incubation in 2 mg/ml L-DOPA in 10 mM Tris buffer pH 8.5 (24h, 30-50°C, pH 5.5-8.5, 10-30 rpm) containing:<br>**Na^+:** 47.22 mM or 472.2 mM or 4.722 M

		**Mg^{2+}**: 5.37 mM or 53.7 mM or 537 mM<br>**Ca^{2+}**: 1.06 mM or 10.6 mM or 106 mM<br>**SO_4^{2-}**: 2.82 mM or 28.2 mM or 282 mM<br>**Cu^{2+}**: 0.05 mM or 0.5 mM or 5 mM<br>2. washing in water 10 times<br>3. drying (37°C), 24 h
P-C-P	**C**oated by PLD with sodium **P**eriodate	1. Incubation in 2 mg/ml L-DOPA in 10 mM Tris buffer pH 8.5 (24h, 30°C, pH 8.5, 30 rpm) containing **$NaIO_4$:** 20 mM<br>2. washing in water 10 times<br>3. drying (37°C), 24 h
P-C-H	**C**oated by PLD with **H**$_2$O$_2$ and Cu^{2+}	1. Incubation in 2 mg/ml L-DOPA in 10 mM Tris buffer pH 8.5 (24h, 30°C, pH 8.5, 30 rpm) containing **H_2O_2** 19.6 mM and **Cu^{2+}** 0.5 mM<br>2. washing in water 10 times<br>3. drying (37°C), 24 h
P-C	**C**oated by PLD	1. Incubation in 2 mg/ml L-DOPA in 10 mM Tris buffer pH 8.5 (24h, 30°C, pH 8.5, 30 rpm)<br>2. washing in water 10 times<br>3. drying (37°C), 24 h
P	Uncoated	1. Incubation in 10 mM Tris buffer pH 8.5 (24h, 30°C, pH 8.5, 30 rpm)<br>2. washing in water 10 times<br>3. drying (37°C), 24 h

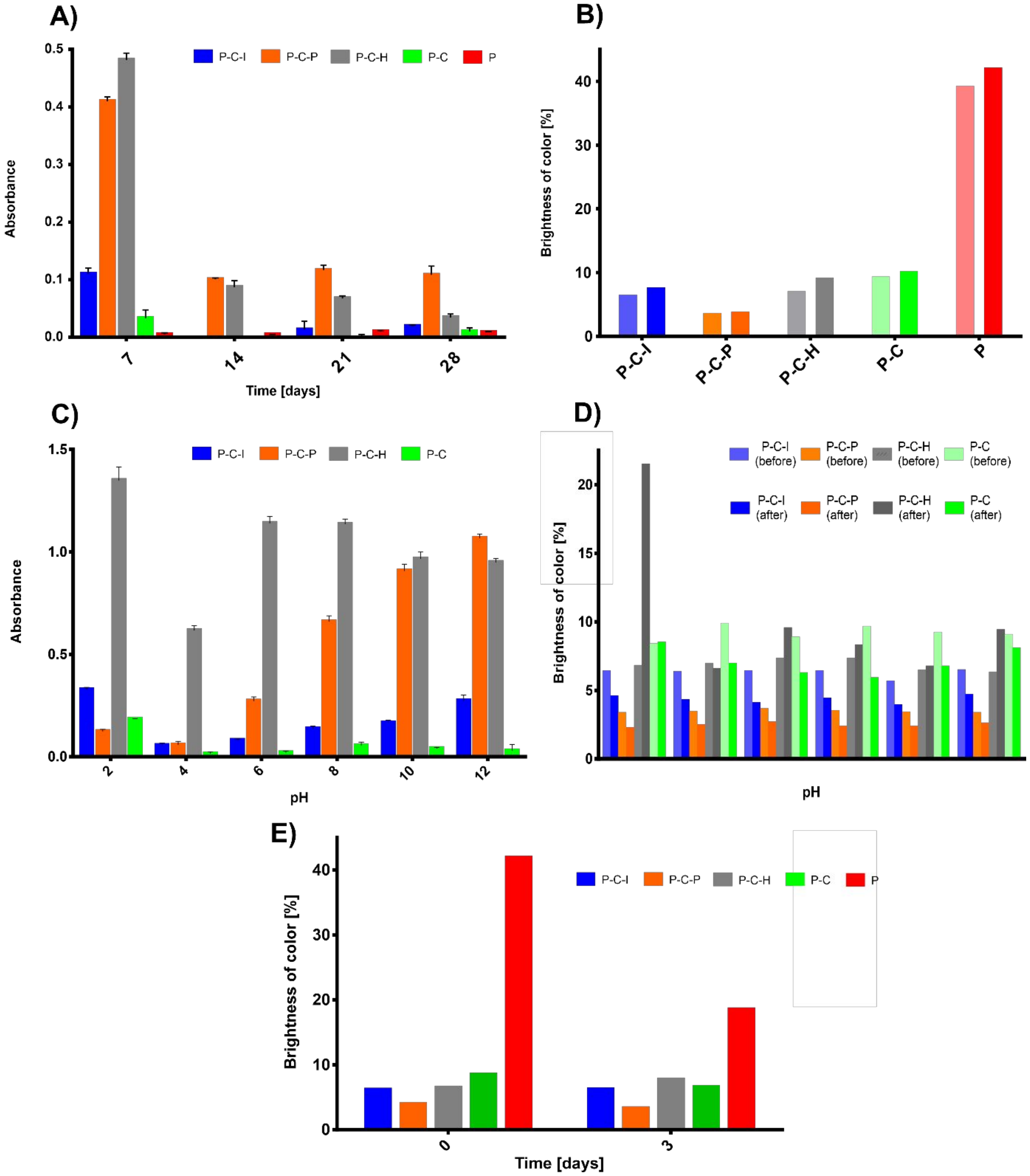

Figure S1. A) Color intensity of SBF incubated with prostheses during the test, as a function of absorbance at 280 nm. **B)** Color intensity of prostheses before and after incubation in SBF, calculated using ImageJ 1.52v. **C)** Color intensity of buffers incubated with prostheses during the test, as a function of absorbance at 280 nm. **D)** Color intensity of prostheses before and after incubation in

buffers, calculated using ImageJ 1.52v. **E)** Color intensity of prostheses before and after incubation with human blood, calculated using ImageJ 1.52v.

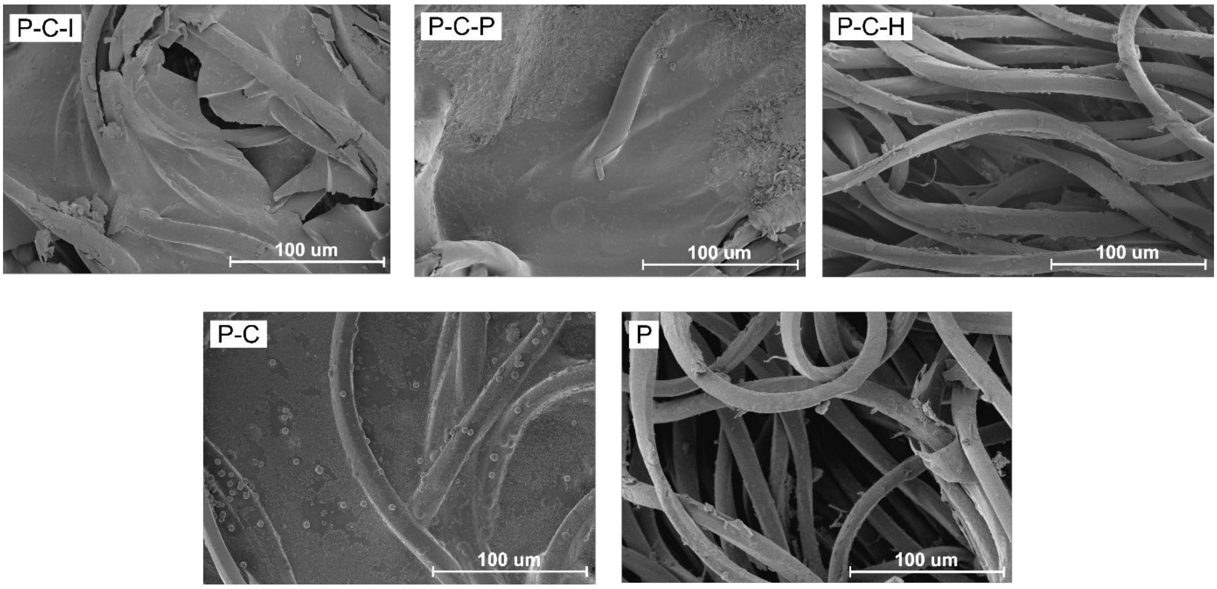

Figure S2. SEM pictures of modified prostheses after 28 days incubation in SBF.

Table S2. Bacterial growth inhibition zones. (G- - without gentamicin; G+ - with gentamicin).

***S. aureus* [mm]**		
	**G-**	**G+**
P-C-I	0	29
P-C-P	0	30
P-C-H	0	29
P-C	0	26
P	0	23
**Control [mm]**		
**Antibiotic concentration [ug]**		
10	27	
50	33	
100	34	
***E. coli* [mm]**		
	**G-**	**G+**
P-C-I	0	28
P-C-P	0	28

P-C-H	0	23
P-C	0	21
P	0	19

Control [mm]

**Antibiotic concentration [ug]**	
10	26
50	31
100	34
